# Human Papillomavirus Infection and Cervical Cancer: Epidemiology, Screening, and Vaccination—Review of Current Perspectives

**DOI:** 10.1155/2019/3257939

**Published:** 2019-10-10

**Authors:** Chee Kai Chan, Gulzhanat Aimagambetova, Talshyn Ukybassova, Kuralay Kongrtay, Azliyati Azizan

**Affiliations:** ^1^Department of Biomedical Sciences, Nazarbayev University School of Medicine, 5/1, Zhanybek and Kerey Khans St., Astana, Kazakhstan; ^2^University Medical Center, National Research Center for Mother and Child Health, Turan Ave., 32, Astana, Kazakhstan

## Abstract

Viral infections contribute as a cause of 15–20% of all human cancers. Infection by oncogenic viruses can promote different stages of carcinogenesis. Among many types of HPV, around 15 are linked to cancer. In spite of effective screening methods, cervical cancer continues to be a major public health problem. There are wide differences in cervical cancer incidence and mortality by geographic region. In addition, the age-specific HPV prevalence varies widely across different populations and showed two peaks of HPV positivity in younger and older women. There have been many studies worldwide on the epidemiology of HPV infection and oncogenic properties due to different HPV genotypes. However, there are still many countries where the population-based prevalence has not yet been identified. Moreover, cervical cancer screening strategies are different between countries. Organized cervical screening programs are potentially more effective than opportunistic screening programs. Nevertheless, screening programs have consistently been associated with a reduction in cervical cancer incidence and mortality. Developed countries have achieved such reduced incidence and mortality from cervical cancer over the past 40 years. This is largely due to the implementation of organized cytological screening and vaccination programs. HPV vaccines are very effective at preventing infection and diseases related to the vaccine-specific genotypes in women with no evidence of past or current HPV infection. In spite of the successful implementation of the HPV vaccination program in many countries all over the world, problems related to HPV prevention and treatment of the related diseases will continue to persist in developing and underdeveloped countries.

## 1. Introduction

According to the World Health Organization's (WHO) statistics, common cancers are one of the most prevalent causes of mortality worldwide with 8.2 million deaths in 2012, and this trend has not changed in recent years. Viral infections contribute to 15–20% of all human cancers, whereby several viruses play considerable roles in the multistage development of malignant cancers. Over the past two decades, it has become obvious that several viruses play an important role in the development of human cancers. Around 15% to 20% of cancer cases are associated with viral infections. Oncogenic viruses can facilitate various stages of carcinogenesis [1]. One of the viruses contributing to the statistics of cancerous diseases is human papillomavirus (HPV). HPV is a virus that can be sexually transmitted, and high-risk HPV DNA is found to be present in 99.7% of cervical cancer specimens [2]. Within 12 to 24 months of exposure to the virus, 90% of HPV infections are cleared or become inactive. However, infections by the high-risk HPV types persist which then increase the risk of progression to cervical cancer [3].

HPV is a double-stranded DNA virus belonging to the Papovaviridae family. Almost 200 HPV types have been identified with more than 40 types colonizing the genital tract. All HPV infection types are divided into two groups based on their carcinogenic properties; these are high risk and low risk. High-risk types include 16, 18, 31, 33, 35, 39, 45, 51, 52, 56, 58, 68, and 59. Others are classified as potential high-risk (which are 53, 66, 70, 73, and 82). Currently, it is well known and proven that HPV16 and 18 are the most virulent high-risk genotypes, causing about 70% of all invasive cervical cancer in the world [4].

At the present time, we have a relatively clear picture of HPV infection's natural history, oncogenic properties, screening, and prevention algorithms. However, HPV infection rates continue to persist, especially in developing countries, where cervical cancer incidence and prevalence are still high. This is due to different reasons, which include low socioeconomic status, lack of population awareness, and inadequately implemented screening and vaccination programs. Thus, it is necessary to continue this discussion and to refocus attention of specialists and population worldwide to HPV infection and related diseases. The aim of this review article is to summarize updated information regarding the aforementioned aspects of HPV infection and related cancers, including also discussions about the HPV genome and molecular events leading to cancer development following an HPV infection. Enhanced knowledge of HPV status and cancer progression events contributes to the improvement of the future management of patients with cervical lesions; this in turn can help mitigate cervical cancer progression among HPV-infected women.

## 2. The HPV Genome

Papillomavirus genome is comprised of a small double-stranded and highly conserved DNA with an approximate size of 8000 base pairs and consists of three regions. The molecular biology of this small DNA molecule is complex. There are six early proteins, three regulatory proteins (E1, E2, and E4) and three oncoproteins (E5, E6, and E7) encoded in 4000 base pairs (bp) that participate in viral replication and transformation of cell. Another 3000 bp region of DNA molecule encodes two structural proteins L1 and L2 that compose the capsid of virus. The viral DNA replication and transcriptional regulatory elements are controlled by a long control region (LCR) that is encoded in a 1000 bp region [5].

Upon the viral evolution, accumulation of numerous lineage-defining genetic variations in these regions can lead to speciation into separate HPV types. Sequence variations such as single-nucleotide polymorphisms or genetic mutations within L1, LCR, E6, and E7 regions of HPV can determine families, relatedness, and phylogeny of the HPV types. HPV type can be defined as an entity based on the more than 10% difference in the DNA sequence of the L1 gene between two genomes. However, the difference between 2% and 10% determines the HPV subtypes. In addition, the variants are entities that define less than 2% of dissimilarities between HPV genomes. According to recent studies, there are 60 out of 160 HPV types associated with mucosal epithelia and categorized as Alphapapillomavirus genus (alpha-PV) [6]. Furthermore, alpha-PV can be classified into nine groups: alpha-5 (HPV23, 51, 69, and 82), alpha-6 (HPV30, 53, 56, and 66), alpha-7 (HPV18, 39, 45, 59, 68, 70, 85, and 97), and alpha-9 (HPV16, 31, 33, 35, 52, 58, and 67), which include mostly the oncogenic high-risk types [7]. However, there are also Betapapillomavirus and Gammapapillomavirus genus that have not been investigated in detail yet [8].

According to Papillomavirus Nomenclature Committee, each HPV type can be differentiated into phylogenetic lineages in terms of geographic distribution, pathogenicity, regulation of transcription, and immunological response [9]. The alpha-9 HPV16 type has been further classified into four phylogenetic lineages: A, B, C, and D. Phylogeny A is divided further into four sublineages A1, A2, A3, and A4. Sublineages A1, A2, and A3 include European HPV DNA sequences while A4 includes Asian sequences isolated worldwide. Lineage B is classified as B1 and B2 sublineages, which comprise the African HPV sequences. Lineage C is also referred to as African sequences. Lineage D consists of 3 sublineages: D1, D2, and D3 that include Asian-American and North American sequences. HPV intratypic molecular variants can be distinguished based on oncogenic potentials in spite of their phylogenetic relatedness. Several research studies associate the HPV16 lineage D as being more tumorigenic in comparison with the other lineages [10].

## 3. Association between HPV Infection and Cervical Lesions

The vast majority of HPV infections are transitory and become undetectable in 12–24 months [4, 11–14]. However, in some women whose infections continue to persist, the risk of developing precancerous conditions is significant. Many studies confirmed that persistent infection with an oncogenic HPV type is the main risk factor for detecting a cervical intraepithelial neoplasia (CIN) that may range from CIN1 to CIN3 and cancer [12, 13, 15]. In the VIVIANE study, the researchers found that HPV33 and HPV16 were associated with the highest risk of CIN development, followed by HPV18, HPV31, and HPV45 [13].

Natural history of CIN lesions is different depending on its grade. CIN1 is a low-grade squamous intraepithelial lesion (LSIL). According to statistical data, over 70–80% of CIN1 lesions spontaneously regress without treatment or become undetectable [11, 16]. Thus, CIN1 reflects a state of infection rather than a stage in disease development. Detection of CIN1 following HPV infection does not therefore automatically represent disease progression. Furthermore, obvious clearance may be attributed to an inability to detect the infection [13]. Therefore, clearance rates should be interpreted with caution.

CIN2 and CIN3 are considered high-grade dysplasia or high-grade squamous intraepithelial lesion (HSIL); however, they are different whereby CIN2 less commonly progresses to cancer. CIN2 develops in two different ways; the annual regression rate of CIN2 in adult women is estimated to range from 15 to 23%, with up to 55% regressing by 4–6 years [16, 17], whereas approximately 2% of CIN2 lesions develop to CIN3 within the same period. CIN3 is considered a true precancer with the potential to progress to invasive cancer at a rate of 0.2% to 4% within 12 months [16, 18]. Untreated CIN3 has a 30% probability of becoming invasive cancer over a 30-year period, although only about 1% of properly treated CIN3 will become invasive [12, 16, 18, 19]. Adenocarcinoma of the cervix is distinct from squamous cell carcinoma as it arises from the glandular epithelium of the endocervical canal and its immediate precursor is adenocarcinoma in situ. The time from HPV infection to cervical cancer development is typically 20 years; therefore, rapid progression of cervical cancers rarely occurs [20].

The link between high-risk HPV types and cervical cancer development contributed to the introduction of novel screening programs. For example, testing for the presence of high-risk HPV is recommended as a screening tool by the WHO and the European Guidelines for Quality Assurance for Cervical Cancer Screening [21, 22]. HPV testing has been found to be effective in detection of precancerous cervical lesions particularly in population-based cervical screening programs [23]. The establishment of the causal link between HPV and cervical cancer, along with an understanding of the epidemiology and natural history of HPV infection, has led to a new model for cervical carcinogenesis: HPV acquisition, HPV persistence, progression to precancer, and invasion [24], which helps guide age-appropriate interventions to prevent cervical cancer.

## 4. Pathogenesis of Cervical Cancer Development following HPV Infection

Cervical cancerogenesis can be defined as the complex mechanism of uncontrolled cellular division that can involve HPV gene integration together with other cellular changes and epigenetic factors. As the HPV infection occurs, the DNA can undergo mutations under the cellular and other environmental conditions leading to viral DNA integration and operation with the host DNA synthesis machinery. As a result, virus can escape cellular and immune defense mechanisms while promoting cell proliferation and inhibiting cellular apoptotic mechanisms.

Oncogenic potential of HPV16 depends on the regulation of viral transcriptional factors. At the initiation of viral infection, the HPV16 genome can be presented as unintegrated small DNA molecule also called episome and results in benign and precancerous lesions of the cervix. However, HPV16 can integrate its genome into the host genome, which in turn can lead to the development of cervical carcinoma and cervical intraepithelial neoplasia grade III [10]. Viral genome integration in combination with dysregulation of the E2 protein, which is a repressor of the oncoprotein, contributes towards the carcinogenic process. These events cause overexpression of E6 and E7 proteins that eventually contribute to viral carcinogenesis by altering cellular apoptotic mechanism [5, 10]. Overexpression of E6 and E7 alone is insufficient to contribute to the cancerogenesis as other genetic and epigenetic factors also need to be established.

**Figure 1:**
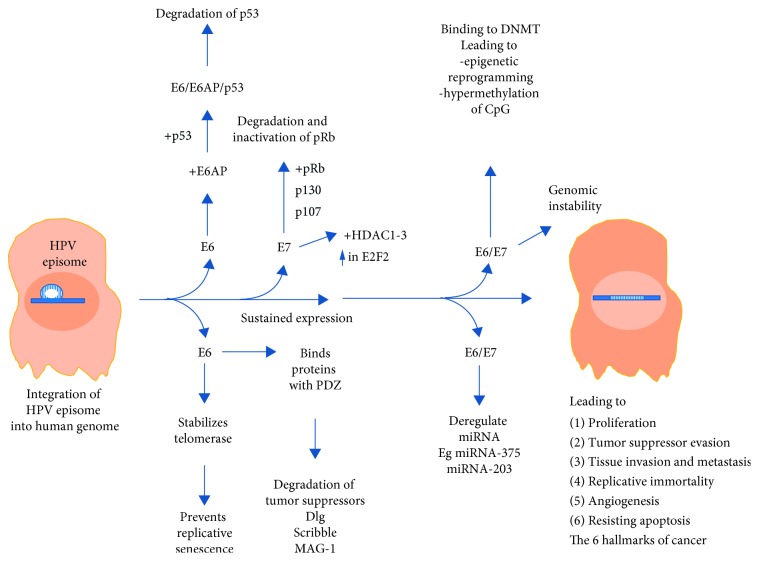
Progression of cervical cancerogenesis which involves HPV gene integration, leading to sustained expression of E6 and E7, impacting and dysregulating the various pathways including the inactivation and degradation of p53 and pRB that lead to uncontrolled cellular division, proliferation, tumor suppressor evasion, and other features of tumorigenicity.

There are many types of HPV, which are found to be associated with cancerous diseases—16, 18, 31, 33, 35, 39, 45, 51, 52, 56, 58, 59, 68, 73, and 82 types [4]. The most carcinogenic HPV type is HPV16, and 50% of all cervical cancers are associated with HPV16 [15]. In HPV16-positive cells, it is found that E6 and E7 viral genes are retained integrated into the host genome and are expressed, although in some HPV16-infected cells E6/E7 overexpression can be absent. Moreover, E6/E7 overexpression is also found in cells infected by other HPV types [25, 26]. E6 and E7 are small proteins of 150 and 100 amino acids without any known enzymatic activity, but they can influence the host cell activity by binding with cellular proteins. E6, for example, binds with E6-associated binding protein (E6AP), a ubiquitin ligase leading to a structural change in E6 allowing it to bind with p53, the cell cycle control tumor suppressor protein to form a trimeric complex E6/E6AP/p53 (Figure 1).

This binding leads to the degradation of p53 and thus leads to cell proliferation. E7, on the other hand, binds pRb causing its inactivation and degradation. Both the low-risk and high-risk E7 protein has been shown to target the pRB family members including p107 (RBL1) and p130 (RBL2) for degradation [27]. pRb downregulates E2F a transcription factor. As pRb is deactivated by E7, E2F is upregulated and cell proliferation genes are activated. Furthermore, E6 and E7 have been shown to form complexes with hundreds of other proteins in the host cell [28–30] and it will be interesting to understand the functions and consequence of what these complexes do. It is important to note that E6 and E7 transforming and oncogenic properties involve other cancer pathways not involving p53 or pRB. For example, E7 stimulates telomerase activity [31] and E6/E7 has been shown to deregulate miRNA linked to carcinogenesis [32]. E7 has also been shown to interact with histone deacetylase- (HDAC1-3-) enhancing E2F activation that is associated with differentiation and viral replication [33].

miRNA plays an important role in the posttranscriptional control of the expression of host genes. Recent studies proposed that HPV E6, E7, and E5 oncoproteins regulate the host miRNA profile. In HPV-associated cervical cancer cells, a number of miRNAs such as miR-21, miR-143, and miR-9 are overexpressed, thus targeting CCL20 (chemokine (C-C) motif ligand) and promoting migration of HPV16-positive cancerous cells. However, overexpression of some miRNAs such as miR-203 inhibits HPV amplification. Thus, in the HPV-infected cancer cells, miR-203 is suppressed by HPV E7 gene overexpression, leading to the induction of viral replication. Deregulation of miRNA expression can occur mostly due to epigenetic methylation of miRNA promoters [34].

E6 belonging to tumorigenic HPV types harbors a PDZ binding motif (PBM) at the C terminus which facilitates the binding of E6 to a number of proteins containing the PDZ site. The binding of E6 to these proteins leads to inactivation and degradation. Examples of such proteins include potential tumor suppressors such as Dlg [35], MAGI-1 [36], and Scribble [37, 38].

The epigenetic control of viral and host gene expression plays an important role in carcinogenesis by involving changes in DNA methylation, modifications of histones, and noncoding RNA profile. Cervical carcinogenesis is strongly associated with persistent HPV infection that can further affect both the host genome and the viral genome methylation process [34].

E6 and E7 have been shown to bind DNA methyltransferases (DNMT) which impairs their activity leading to hypermethylation of CpG islands which can eventually lead to possible silencing of host tumor suppressors [30, 39]. Some studies showed decreased methylation of the upstream regulatory region (URR) in the cervical cancer cells in comparison with normal cells, whereas other studies described increased methylation of the viral genome [40]. These studies' discrepancies can be explained by the viral life cycle stages, type of HPV genome integrations, cervical cancer stages, and other factors. However, methylation of viral DNA can be defined as the host cellular defense mechanism. So, it is still poorly investigated if HPV DNA methylation is beneficial for viral cancerogenesis [34].

It has been suggested that increased methylation of CpG dinucleotides within E2 binding site (E2BS) on the host genome can modify interaction of different factors and result in abnormal cell differentiation with further disease progression [34]. As a result, this hypermethylation event reduces the binding affinity of the viral regulatory protein E2 to E2BS, thus leading to E6 and E7 overexpression and further epigenetic inhibition of tumor suppressor genes [10]. Some studies suggest that CpG region methylation can be used as a biomarker of cervical cancer detection.

## 5. Epidemiology of Cervical Cancer

Cervical cancer is the leading genital cancer among women worldwide, with almost half a million new cases per year (GLOBOCAN, 2012) [41]. In 2015, 526,000 women developed cervical cancer worldwide and caused 239,000 deaths [42]. The majority of cervical cancer cases are squamous cell carcinoma [41]. In spite of effective screening methods, cervical cancer continues to be a major public health problem [4].

The mortality from cervical cancer varies in different geographic regions. The age-standardized incidence rate for cervical cancer is much lower in developed countries at 5.0 per 100,000 compared to developing countries at 8.0 per 100,000 [43]. Similarly, the age-standardized mortality rate for cervical cancer is lower in developed nations at 2.2 per 100,000 compared with developing nations at 4.3 per 100,000. For example, in sub-Saharan Africa, there were 34.8 new cases and 22.5 deaths per 100,000 women, while in Western Asia there were only 4.4 new cases and 1.9 deaths per 100,000 women in 2012 [44]. In comparison, Northern America is found to be the region with the third-lowest cervical cancer rate in the world [43].

Limited statistical data are available on cervical cancer in Central Asia [43]. From the existing sources, it is found that the incidence rates of cervical cancer in many countries of Central Asia are quite high (ranging from 9.9 per 100,000 women in Tajikistan to 29.4 per 100,000 in Kazakhstan) compared to Europe (ranging from 4.0 per 100.000 in Finland and 7.0 per 100.000 in Germany) [43–45]. Approximately 25,700 women are diagnosed with cervical cancer and 12,700 die from this disease annually in the Central Asian countries [46]. The mortality rates range from 4.9 per 100,000 women in Tajikistan to 11.2 per 100,000 in Kyrgyzstan [41, 46]. The indicators are higher than in Western European countries (incidence rates ranging from 2.1 per 100,000 women in Malta to 12.2 per 100,000 in Portugal; mortality rates ranging from 0.8 per 100,000 women in Iceland to 3.6 per 100,000 in Portugal) [46].

Cervical cancer has a bimodal age distribution with the majority of cases occurring among women in their 30s and 40s, the age at which women are often raising families and ensuring the financial viability of their families and communities. In addition to the risk of death, cervical cancer is associated with increased morbidity, including bleeding, pain, and kidney failure, which are difficult to treat, especially in communities with poor access to healthcare [47].

## 6. Prevalence of HPV in the General Population and in Cervical Cancer Patients

HPV infections are widespread all over the world; however, prevalence and type distribution are heterogeneous [48]. The age-specific HPV prevalence varies in young and advanced age women populations [49]. A comprehensive meta-analysis assessing the global prevalence of cervical HPV infection among women without cervical lesions revealed that almost 12% of females worldwide are positive for HPV DNA [50].

There have been many studies worldwide on the epidemiology of HPV infection and oncogenic properties due to different HPV genotypes [4]. One of the international studies found that 10.4% of patients with normal cytology have been detected with either high- or low-risk HPV types. Women in less developed countries and those who are younger than 25 years old have a higher prevalence, ranging from 15 to 45% [50]. The highest HPV prevalence was observed in sub-Saharan Africa (24%), Eastern Europe (21.4%), and Latin America (16.1%) and the lowest in Northern America (4.7%) and Western Asia (1.7%). The HPV type 16 was the most common virus worldwide with prevalence rates accounting for 32.3% of all infections in Southern Asia, 28.9% in Southern Europe, 24.4% in Western Europe, 24.3% in Northern America, and 12% in Africa [51].

According to the Extended Middle East and North Africa (EMENA) study, in the Middle East, the incidence of HPV shows lower rates compared to the rest of the world [52]. For instance, in Qatar HPV prevalence among the general population of women with normal or abnormal cytology recently estimated 6.1% [52]. The authors detected the presence of various HPV genotypes with a high prevalence of low-risk HPV types, particularly type 81.

Very limited data are available on HPV prevalence, incidence, and genotype-specific dissemination in Central Asia and Eastern Europe. For example, according to the report of HPV Information Centre (2017), no data on the epidemiology of HPV infection are available in Kazakhstan (which is a Central Asian country), and only a few articles on the epidemiology of HPV infection in Kazakhstan were published in international peer-reviewed journals and several articles in local medical journals [53]. The authors' findings demonstrated that 43.6% of the patients attending gynecologic clinic were HPV positive. The most prevalent types detected were HPV16 (18.4%) and HPV18 (9.22%), followed by HPV types 33, 51, and 52 (nearly 5% each) [53].

The prevalence of HPV infection among Africans is higher than in the European population with 26.3% in Nigeria, 47.9% in Guinea, 41% in South Africa, and 38.8–42.3% in Kenya [54, 55]. Possibly high prevalence of HPV among women in sub-Saharan African countries is more prominent due to high exposure of human immunodeficiency virus (HIV) in the country, and cervical cancer may become epidemic if cervical cancer knowledge is not increased and the barriers for early screening services will still exist [56].

Other studies highlighted that some special populations have a higher risk of acquiring HPV infection. A study investigated the prevalence of HPV infection among the adolescent population in Uganda has shown significantly high distribution of high-risk HPV types (16, 18, 31, 52, and 58), which is 51.4% [57]. The reasons for such high prevalence were explained by sexual behavior, which includes early age of sexual debut and multiple sexual partners. Those factors put young women at higher risk of HPV infection [50].

With the development of highly sensitive HPV DNA testing, studies have confirmed that most cervical cancer specimens have detectable HPV DNA, and greater than 90% contain DNA for HPV16, 18, 31, 33, 39, 45, 52, or 58 [7]. It should be noted that women who develop cervical cancer have often had the same type of high-risk HPV detected in cervical specimens 3 to 5 years prior to their cancer incidence. Unfortunately, HPV genotyping can only detect current infection; therefore, we are not able to understand when in the lifetime HPV has had a carcinogenic effect [58].

Some investigators have identified regional differences in the prevalence of squamous cell carcinoma linked to HPV infection. In a meta-analysis of 85 studies, which included 10,058 women with cervical cancer, HPV16 prevalence predominated in squamous cell carcinoma, ranging from 46% in Asia to 63% in North America. The second most prevalent type was HPV18, found in 10–14% of squamous cell carcinoma specimens. The frequency of adenocarcinoma among all invasive cervical cancers also remains significant. It ranges from 4% in Africa to 32% in North America. As expected, high-risk HPV type 18 was found to be dominant in adenocarcinoma cases with a prevalence that ranges from 37% to 41%. The next most common HPV types are type 16 and type 45, which were found in 26–36% and 5–7% of samples, respectively [50]. According to the meta-analysis that included 133 studies and 14,595 women, combination of HPV16 and 18 contributes to 74–77% of squamous cell carcinoma in Europe and North America, and 65–70% of squamous cell carcinoma in Africa, Asia, and South/Central America [50]. While data from meta-analyses are limited by their reliance on the HPV DNA testing methods of each individual study, multiple studies collecting samples from large cohorts have confirmed the presence of the same HPV types in invasive cervical cancer specimens.

Several international studies investigated the prevalence of HPV types in invasive cervical cancer specimens. One of those studies explored the most prevalent types in 1918 women with cervical cancer. For that purpose, cervical cancer cells were directly tested for HPV types and the researchers found the following HPV types to be the most prevalent: HPV16, 18, 45, 31, 33, 52, 58, and 35 [59]. Similarly, an international study conducted in 38 countries tested invasive cervical cancer paraffin block samples from 10,575 women for the presence of certain HPV types. The researchers found HPV DNA in 8977 of the samples, which comprise 85% of all specimens. HPV16 or 18 was detected in 71%, and types 31, 33, 35, 45, 52, and 58 were detected in an additional 20% of the HPV-positive samples.

Having a high incidence and mortality from cervical cancer makes the screening program very important. Enhancing public awareness of underlying causal factors is a high priority for developing an appropriate cancer control and prevention program.

## 7. Cervical Cancer Screening

It is well known that cervical cancer screening can reduce cervical cancer incidence and mortality [60]. Cervical cancer screening strategies are different between countries. Some countries have population-based programs, whereby women in the target population are individually identified and invited to attend the screening. In opportunistic screening, invitations depend on the individual's decision or on encounters with healthcare providers. Organized cervical screening programs may achieve high participation at regular intervals with equal access, and high-quality standards for diagnosis, thus potentially more effective than opportunistic screening [61, 62]. Examples of organized programs for cervical cancer screenings exist in high-income countries such as the United Kingdom, Australia, Canada, Finland, the Netherlands, and Singapore. On the other hand, Eastern European countries have an opportunistic screening with lower‐screening coverage and lower‐immunization coverage and show high cervical cancer incidence and mortality rates [42]. In most of the Central Asian countries, the Caucasus region, the Russian Federation, and the Western countries of the former Soviet Union, cervical cancer screening is mainly opportunistic and characterized by cytology testing, using Romanowsky staining and generally low or unreported coverage [63]. Nevertheless, cervical cancer screening contributes to a decrease in cervical cancer incidence and mortality [64].

HPV vaccine was introduced later. Developed countries have accomplished reduction of cervical cancer incidence and mortality during the last 40 years due to the introduction of cytological smear screening [65]. For instance, since the introduction of the Pap smear cytology testing in the 1950s and 1960s, cervical cancer incidence and mortality have declined in the United States with organized cervical cancer screening programs and screening rates of 83% [66]. In the Northern European countries, an organized screening program was established in the 1960s and their effects on cervical cancer incidence and mortality have been accurately investigated [62]. However, in the greater part of Europe, evaluation systems are insufficient and nonstandard.

At the same time, cervical cancer prevalence remains at a high level in developing countries of Central and South-East Asia, Africa, and Eastern Europe, where cervical cancer screening programs are not properly implemented due to a variety of reasons (socioeconomic, geographic, etc.). Population coverage by screening program in developing countries ranges between 6 and 8% [67]. In recent years, international recommendations for screening have been developed to include HPV testing, where available [68]. Despite marked advances in knowledge about cervical cancer and effective screening, cervical cancer screening programs have variable efficacy depending on availability of resources, implementation strategies, quality of laboratory and pathology testing, and community awareness [69]. Effective cytological screening of cervical specimens and HPV genotyping require materials and specialists that are complicated and expensive for many low-income countries [70]. Even in developed countries with advanced healthcare systems and long-standing cervical cancer screening modalities, population coverage is not perfect [71].

There is also discrepancy in the frequency of the screening tests among countries and age groups [72]. In developed countries like England and the USA, screening is scheduled every 3 years for women aged 21–29; starting from 30 years old until 65 years old, the screening tests are recommended for every 5 years [73–75]. Results of the population-based survey of adults aged 50–70 in England suggest that although awareness of the purpose of early detection screening is high, awareness that screening can prevent cancer is low across all demographic groups [74].

In most of the developing countries of Africa, Central Asia, South-East Asia, Eastern Europe, screening is scheduled every 5 years or even rarer [72, 76]. However, there are several exclusions. For instance, in Kyrgyzstan, republic of Central Asia, there is no cervical cancer screening program at all [63]. In South Africa, a national cervical screening policy was formulated in 2000 and allowed for three free cervical smear tests, conducted at 10-year intervals from the age of 30 years [72]. This policy has been implemented in some areas; however, there is currently no population-wide screening program in South Africa.

Although the recommended screening modalities for cervical cancer have contributed to a reduction in cervical cancer incidence and mortality due to cervical cancer, the benefits of cervical cancer screening are yet to be fully realized in countries with poorly organized screening programs for women at risk. The updated WHO recommendations for cervical cancer screening and prevention are summarized in Table 1 [21].

It is also noteworthy that even in countries with organized screening services, these benefits are not maximized in underserved, uninsured, and underrepresented populations due to factors such as cost, access problems, anxiety, discomfort with the screening procedure, and fear of cancer or poor health literacy, all of which contribute to poor outcomes for cervical cancer [77].

Incorporation of HPV testing into cervical cancer screening strategies has the potential to allow both increased disease detection and increased length of screening intervals (decreasing harms such as psychosocial impact of screening positive, additional clinical visits and procedures, and treatment of lesions destined to resolve).

## 8. HPV Vaccination

Statistical data from the recent years show that utilization of HPV vaccines is very effective for preventing infection and disease related to the specific HPV genotypes [78]. Vaccination programs have been very successfully implemented in many countries all over the world [78, 79].

There are three commercially prophylactic vaccines available; these are Cervarix (a bivalent vaccine against HPV16 and HPV18), Gardasil (a tetravalent against HPV6, 11, 16, and 18), and Gardasil 9 (9-valent vaccine against HPV6, 11, 16, 18, 31, 33, 45, 52, and 58). They are noninfectious subunit vaccines containing viral-like particles (VLP) derived from the assembly of the recombinant expression of L1 major capsid protein of the HPV in yeast (Gardasil) and in insect cells (Cervarix). Administration of the vaccine is carried out by intramuscular injection with three doses of prime/boost series over a 6-month period. Early analysis shows that even a single dose can reduce infection and is effective in preventing the persistent incidence of infection and premalignant neoplasia [80]. The exceptionally strong and lasting antibody response has been well documented; for example, the 100% seroconversion rate in young healthy women, preadolescent boys, and girls with antibody response remains stable for over a decade [81]. The exact molecular mechanism however is still elusive in humans as HPV hosting organism, and presently, there is no human model to study the mechanism except on transgenic mouse models and xenograft models [38]. Screening remains to be the only form of prevention for 2 to 3 generations of women beyond the adolescent target age for vaccination [82].

At the present time, we have an abundance of evidence from multiple countries, with a different level of HPV vaccination coverage and implementation strategies that show the vaccines are effective [78]. In developing countries with long-standing screening programs, catch-up vaccination cohorts and established registration have demonstrated reductions in the diagnosis of CIN in screening women due to vaccination [78]. For example, the researchers from Scotland show a reduction of low- and high-grade CIN associated with high uptake of the HPV bivalent vaccine at the population level [83]. Results from one of the recent studies from Japan demonstrated that women aged 20–24 years who received HPV vaccination had significantly lower rates of abnormal cervical cytology results when compared to those who did not receive the vaccine [79]. An Australian study found that vaccination employing tetravalent HPV vaccine helps to reduce cases of HSIL and LSIL in females [84]. Research findings from Canada suggest that the HPV vaccination was moderately effective in preventing HSIL among adolescents but far less effective in the older age groups, especially among those with a history of abnormal cytology [85].

Taking into consideration the present efforts to increase HPV vaccinations for primary cervical cancer prevention, early detection of precancerous cervical lesions through screening remains to be very important in order to timely diagnose and reduce cervical cancer incidence and mortality. This is especially true in low-income regions where HPV vaccination has not yet implemented and supported at the governmental level [86]. Developed countries, with well-established cervical cancer screening programs, have achieved an impressive reduction in cervical cancer incidence and mortality, while developing countries with lack of HPV vaccination and/or worse modalities of screening programs still have a high level of adverse outcomes [87]. These discrepancies in HPV vaccination envelopment could explain the differences in incidence, prevalence, and mortality linked to cervical cancer in different countries in the world.

HPV vaccination for the prevention of high-risk HPV types is expected to reduce cervical cancer burden [88]. Supporting HPV vaccines' effectiveness against cervical cancer is difficult due to the long period between initial infection and cancer development. Surrogate markers therefore have been proposed to determine vaccines' effectiveness on a shorter term, such as population-based continuous monitoring of high-grade precursor lesions such as CIN3 [89]. Statistics received from studies that covered large cohorts of women after implementation of Cervarix or Gardasil have shown that both vaccines are effective in order to reduce the frequency of precancerous lesions associated with the vaccine genotypes [90]. On the other hand, even the nonavalent Gardasil vaccine cannot prevent all cervical cancer cases due to type specificity and time of implementation.

There are also apparent limitations and the public health challenges in attempting to implement HPV vaccination programs. These limitations and challenges include the vaccine's type specificity, required to be given prior to exposure, the three-dose schedule, ethical issues in targeting age group of early adolescence, and potential communication challenges around HPV being a sexually transmitted infection [78]. Therefore, large groups of women in advanced age who have not received vaccination are still under the risk of cervical cancer development. Furthermore, HPV screening and vaccination being complementary preventive options are often implemented as separate and noncoordinated public health programs. Therefore, to address this inaccuracy, the recently created “HPV FASTER” protocols aim at combining both strategies with the purpose of accelerating the reduction of cervical cancer incidence and mortality, making the programs both cost-effective and sustainable [91]. The proposal of “HPV FASTER” protocol is to offer HPV vaccination to women in a broad age range of 9 to 45 years irrespective of HPV status.

In developing countries, reduction of cervical cancer incidence and mortality could be achieved only with governmental guidance by the implementation of sustainable and effective screening and vaccination programs.

## 9. Conclusion

Cervical cancer is associated with considerable morbidity and mortality all over the world. It is well known that one of the main causative agents for cervical cancer is high-risk HPV strains, and this type of malignancy is preventable. High incidence of cervical cancer with considerable mortality is an evidence of HPV infection abundance with the absence of the HPV screening and low public awareness of the problem. Substantial incidence and mortality from cervical cancer make the screening program very important. Enhancing public awareness of underlying causal factors is a priority that should be emphasized for prevention programs. Incorporation of HPV testing into screening strategies has a high potential to decrease morbidity and mortality from cervical cancer. The knowledge of HPV prevalence and type distribution could contribute to the successful vaccination program implementation. The educational health promotion projects for the population should be provided to reinforce the knowledge and conversance of this public health problem. From the review given here, it is clear that the HPV screening along with the vaccination program should be implemented and supported at a governmental level in developing countries with high incidence and mortality of cervical cancer.

## Figures and Tables

**Table 1:** WHO recommendations on cervical cancer screening and prevention in the low- and middle-income countries.

	Primary prevention: vaccination		Secondary prevention: screening
Methods	Inclusion of HPV vaccine in the national immunization schedule: (i) Bivalent (ii) Tetravalent		(i) Cervical cytology (conventional Pap smear and liquid-based) (ii) Visual inspection with acetic acid (VIA)/visual inspection with Lugol's iodine (VILI) (iii) HPV testing for high-risk HPV types (i.e., types 16, 18,31, 33, 45, and 58)

Target age group (years) and gender	Girls 9–14 years old	Girls over 15 years old	Women 30–49 years old

Frequency and intervals	2 doses	3 doses	(i) Once in life time (ii) Once in 10 years (iii) Once in 5 years (a) VIA/cytology every 3- to 5-year interval; (b) HPV testing minimum every 5-year interval
	6-month interval	Bivalent: 0, 1, 6 months; tetravalent: 0, 2, 6 months	

Programmatic consideration	School-based delivery strategy		(i) Organized program (ii) Unorganized/opportunistic/sporadic initiatives (a) Screen-and-treat approach (b) Screen-diagnose-treat approach

## Abbreviations

WHO: World Health Organization
HPV: Human papillomavirus
DNA: Deoxyribonucleic acid
CIN: Cervical intraepithelial neoplasia
LSIL: Low-grade squamous intraepithelial lesion
HSIL: High-grade squamous intraepithelial lesion
DNMT: DNA methyltransferases
EMENA: Extended Middle East and North Africa
Pap test: Papanicolaou test
VIA: Visual inspection with acetic acid
VILI: Visual inspection with Lugol's iodine.
